# Implications of MicroRNAs in the Treatment of Gefitinib-Resistant Non-Small Cell Lung Cancer

**DOI:** 10.3390/ijms17020237

**Published:** 2016-02-15

**Authors:** Thomas K. Sin, Fengfeng Wang, Fei Meng, S. C. Cesar Wong, William C. S. Cho, Parco M. Siu, Lawrence W. C. Chan, Benjamin Y. M. Yung

**Affiliations:** 1 Department of Health Technology and Informatics, The Hong Kong Polytechnic University, Hung Hom, Kowloon, Hong Kong, China; Ka.Wai.Thomas.Sin@uth.tmc.edu (T.K.S.); wangff0223@163.com (F.W.); fei.meng@polyu.edu.hk (F.M.); cesar.wong@polyu.edu.hk (S.C.C.W.); parco.siu@polyu.edu.hk (P.M.S.); ben.yung@polyu.edu.hk (B.Y.M.Y.); 2 Department of Integrative Biology and Pharmacology, The University of Texas Health Science Center, Houston, TX 77030, USA; 3 Department of Clinical Oncology, The Queen Elizabeth Hospital, Kowloon, Hong Kong, China; chocs@ha.org.hk or williamcscho@gmail.com

**Keywords:** EGFR, gefitinib, miRNA, non-small cell lung cancer, resistance

## Abstract

Non-small cell lung cancer (NSCLC) represents about 85% of the reported cases of lung cancer. Acquired resistance to targeted therapy with epidermal growth factor receptor-tyrosine kinase inhibitors (EGFR-TKIs), such as gefitinib, is not uncommon. It is thus vital to explore novel strategies to restore sensitivity to gefitinib. Provided that microRNAs (miRNAs) negatively regulate their gene targets at the transcriptional level, it is speculated that miRNA mimetics may reduce the expression, activity and signal transduction of EGFR so that sensitization of tumour sites to gefitinib-induced cytotoxicity can be achieved. Indeed, a growing body of evidence has shown that the manipulation of endogenous levels of miRNA not only attenuates the EGFR/PI3K/Akt phosphorylation cascade, but also restores apoptotic cell death in *in vitro* models of experimentally-induced gefitinib resistance and provoked tumour regression/shrinkage in xenograft models. These data are in concordant with the clinical data showing that the differential expression profiles of miRNA in tumour tissues and blood associate strongly with drug response and overall survival. Furthermore, another line of studies indicate that the chemopreventive effects of a variety of natural compounds may involve miRNAs. The present review aims to discuss the therapeutic capacity of miRNAs in relation to recent discoveries on EGFR-TKI resistance, including chronic drug exposure and mutations.

## 1. Introduction

Non-small cell lung cancer (NSCLC) makes up about 85% of the reported cases of lung cancer. Approximately 10% of NSCLC patients manifest mutations of epidermal growth factor receptor (EGFR), resulting in hyperactivation of downstream oncogenic pathways related to cell proliferation and survival [1]; hence pointing out a therapeutic opportunity through the pharmacological blockade of EGFR. Unfortunately, the emergence of resistance to targeted therapy with epidermal growth factor receptor-tyrosine kinase inhibitors (EGFR-TKIs), such as gefitinib, has been found in a considerable number of patients with repeated therapeutic cycles [2]. Currently, there is no effective approach to reverse the mutations associated with drug resistance, and the inhibition of EGFR alone appears to be insufficient to produce promising clinical outcomes. It is thus necessary to explore novel pharmacological strategies to deactivate the EGFR signalling pathway at multiple levels.

Mature microRNAs (miRNAs) are single-stranded, non-coding RNAs composed of 19–22 nucleotides which have tremendous impact on the transcriptome. Activation of miRNA involves the “trimming” of a primary transcript consisting of hundreds of nucleotides through the action of Drosha, which is a RNA-specific endonuclease, to premature pre-miRNA that is approximately 80 nucleotides in length. Furthermore, pre-miRNA is subject to modification by Dicer protein in the cytoplasm as double-stranded miRNA, but only one of the strands would eventually incorporate with the RNA-induced silencing complex (RISC). Provided that miRNAs induce transcriptional repression through complementary pairing of the 3′-untranslated region (UTR) with the transcripts of interest, it is plausible to postulate that the introduction of miRNA mimetics may reduce the expression, activity and signal transduction of EGFR such that the cytotoxicity of gefitinib can be restored in tumours exhibiting impaired drug response [3]. This review aims to support efforts to improve the therapeutic effects of miRNAs through the discussion of (1) the benefits and limitations of combined treatment with gefitinib; (2) recent investigations of putative actions of miRNAs; and (3) potential synergistic effects with natural compounds.

## 2. Overcoming Gefitinib Resistance: Not Only an Issue of EGFR

Reversible EGFR-TKIs, such as gefitinib and erlotinib, are considered the frontline treatment for advanced NSCLC patients harbouring EGFR mutations. Unfortunately, the therapeutic efficacies of EGFR-TKIs are known to be impeded by mutations of EGFRs. Activating mutations, such as deletions in exon 19 and amino acid substitutions in exon 21 including the well-documented L858R point mutation, elevate the intrinsic tyrosine kinase activity of EGFRs, an effect which establishes the rationale of treatment with EGFR-TKI to reduce competitive binding of adenosine triphosphate (ATP) [4]. Notably, these mutations have been clinically proven evident in NSCLC specimens of gefitinib-responsive patients [1]. Insertions in exon 20 and T790M missense mutation, however, are thought to be early genetic events which confer EGFR-TKI resistance in NSCLCs [5]. It is thought that the threonine-to-methionine substitution at residue 790 would increase steric hindrance at the ATP-binding pocket of EGFR, thereby preventing the binding of gefitinib while favouring the docking of ATP [6]. Structural analyses have revealed that the ATP-binding domain was unaltered in D770_N771insNPG subtype, which is one of the exon 20 insertion mutants exhibiting active kinase activity [7]. Intriguingly, erlotinib mitigated the phosphorylation levels of EGFR^Thr1068^, Akt^Ser473^ and MAPK^Thr202^ and augmented apoptotic cell death measured as Bim and cleaved PARP in BID007 cells harbouring the A763_Y764insFQEA variant of exon 20 [7]. This observation is in line with the clinical data that A763_Y764insFQEA-positive patients manifested drug response to erlotinib [7], although the underlying mechanisms accounting for these conflicting findings are not known.

The phenomenon of EGFR-TKI resistance has called for intense efforts in search of novel, alternative therapeutic opportunities. The fact that T790M mutation increases recruitment and binding of ATP but not EGFR-TKIs and is found in 68% of NSCLC patients with defective responses to EGFR-TKIs [8] have strived for the exploration of irreversible-EGFR-TKIs such as afatinib. Nevertheless, given the role of EGFRs in the maintenance of skin and mucosal health, it is not unexpected that irreversible inhibition of EGFR would be associated with more severe off-target effects. It was reported that grade 3 rash and diarrhoea were prominent in patients exposed to afatinib therapy compared to those treated with erlotinib (afatinib *vs.* erlotinib: rash: 28% *vs.* 13%; diarrhoea: 22% *vs.* 5%) [9,10]. A recent retrospective analysis has reported that afatinib did not confer significant benefits to T790M-positive patients, and the overall survival of these patients was found to diminish further upon concomitant harbouring of exon 19 deletions compared with L858R mutations [11]. These results raise the need for the identification of molecular targets that would be of higher therapeutic value. Targeting vascular endothelial growth factor (VEGF) by the monoclonal antibody bevacizumab was observed to achieve 81%, 66% and 57% inhibition of tumour growth in H157, H460 and A549 xenografts respectively [12]. These data coincided with the amount of VEGF secretion *in vitro* (H157 > H460 > A549), thereby suggesting that up-regulation of VEGF may represent a pathogenic mechanism that contributes to the resistance to EGFR-TKIs [12]. Phosphatidylinositol-3-kinase (PI3K) and mammalian target of rapamycin (mTOR) are 2 upstream molecules known to activate the protein kinase B (Akt) phosphorylation cascade. Simultaneous repression of the PI3K/mTOR axis by NVP-BEZ235 was reported to ameliorate growth and migration of gefitinib-resistant H1975 cells and induce tumour shrinkage in H1975-bearing mice [13]. It is also noteworthy that the immunoreactivities of VEGF and cluster of differentiation 31 (CD31) were blunted concurrently in NVP-BEZ235-treated H1975 tumours compared with the untreated counterparts [13]. Provided that H1975 is an *in vitro* model of gefitinib resistance harbouring both L858R and T790M mutations, researching VEGF effectors may have important translational implications in clinical oncology.

It has also been proposed recently that disruptions of mitochondrial function by oxidative stress may modulate gefitinib resistance. Chronic exposure to gefitinib reduced mitochondrial number and respiration and up-regulated remarkably vimentin, a marker indicative of drug resistance in H1650 cells whereas these alterations were reversed by mTempo, which is a free radical scavenger [14]. Under aerobic conditions, pyruvate dehydrogenase (PDH) is essential for the conversion of pyruvate, which is a glycolytic metabolite, into acetyl-CoA prior to the entry of the Kreb Cycle. Importantly, intracellular production of reactive oxygen species (ROS) and protein expression of E1α/β and E3bp subunits of PDH were elevated and attenuated respectively, in gefitinib-resistant H1650 clones relative to their parental counterparts [14]. Although the linkage between increased oxidative stress and acquisition of drug resistance is lacking, the data point to the notion that augmentation of mitochondrial function by antioxidants may have preventive/therapeutic values in gefitinib-resistant NSCLCs.

## 3. Does Combination Treatment Enhance the Therapeutic Capacity of EGFR-TKIs?

Emerging evidence suggests that the anti-tumour activity of EGFR-TKIs in resistant NSCLC cell lines can be enhanced by combined therapy with other regimens. Early efforts have shown that cetuximab, which is an EGFR-targeting monoclonal antibody, produced synergistic anti-proliferative effects in various tumour cell lines including H226 when used in combination with gefitinib or erlotinib [15]. Further analyses with SCC-1, which is an *in vitro* model of head and neck tumour, uncovered that apoptotic activation and repression of phosphorylated EGFR/Akt/MAPK were more pronounced in the cetuximab plus gefitinib group compared with the gefitinib-treated group [15]. These data are in agreement with a recent attempt showingthat concomitant use of bevacizumab and erlotinib reduced tumour growth remarkably by more than 85% in H157 xenografts relative to less than 40% only in littermates treated with erlotinib alone [12]. Compared with PC9 and HCC827 cells of which also possess deletion of exon 19, the reduction of phosphorylated EGFR^Thr1135^ in H1650 was not paralleled by elevated cleavages of caspase 3 and PARP in response to gefitinib treatment [16]. Simultaneous blockade of IGFR (insulin growth factor receptor) by AG1024, on the contrary, increased the contents of cleaved caspase 3 and PARP and induced apoptotic cell death in H1650 cells [16]. These data are in support of a recent study exhibiting that gefitinib decreased tumour volumes in H1975-implanted mice only when administered in combination with NVP-BEZ235, which is a dual inhibitor of PI3K/mTOR [13].

Another conflicting line of evidence suggests that dual treatment may not necessarily followed by synergistic therapeutic effects. While substantial growth delay and down-regulation of proliferating cell nuclear antigen (PCNA) in H226 tumours were more evident in athymic mice subject to combined therapy with cetuximab and gefitinib/erlotinib relative to their counterparts receiving single treatment with either of the drugs studied [15], these results were not reproduced clinically. Co-administration with cetuximab and erlotinib failed to elicit significant radiographic responses in metastatic lung adenocarcinoma patients with T790M mutation, thus implying that alleviating EGFR activity by current approaches may not be sufficient to confer significant treatment outcomes [17]. In a recent study, suppression of A549 tumour growth by bebacizumab and erlotinib was not more effective than that by erlotinib alone, which was attributable to the low expression of VEGF in the tumour tissues concerned [12].

## 4. Therapeutic Potential of MicroRNAs to Revert Gefitinib Competence

MicroRNAs (miRNAs) have gained increasingly attentions from researchers worldwide due to its known transcriptional silencing effects by which may modulate multiple signalling cascades. While the centre of attention of the current work is to review miRNA regulation in gefitinib-resistant NSCLC, further readings are recommended for readers interested in the implications of miRNAs in other cancer types [18] and recent attempts uncovering the roles of miRNAs in resistance mechanisms of commonly prescribed chemotherapeutics in lung cancer [19]. Studies involving miRNA interventions to attenuate EFGR-TKI resistant phenotypes are encouraging. Although most of them did not elucidate whether the observable effects (augmentation of apoptosis, *etc.*) were due to direct degradation of the target of study or secondary to the repression of other regulatory molecules, inactivation of EGFR/MET appears to be a common mechanism of various miRNAs (Table 1).

**Table 1:** Recent studies on putative actions of various miRNAs.

miRNAs	Models of EGFR-TKI Resistance	Molecular Targets	References
miR-34a	1. HGF/gefitinib-treated HCC827 2. xenografts	↑ PARP cleavage ↓ p-MET/MET ↓ p-EGFR	[20]
miR-7	1. A549	↓ EGFR	[21]
miR-146a	1. H1975 2. Human NSCLC	↑ Caspase 3/7 activity ↓ p-EGFR/EGFR ↓ p-Akt	[22]
miR-9-3	1. H1975	↑ Caspase 3 activity ↑ DNA fragmentation	[23]
miR-210	1. A549	↓ OXPHOS ↑ HIF-1α	[24]
miR-150	1. Human lung cancer samples 2. A549/H1975	↓ SRCIN1	[25]
miR-21	1. PC9 xenografts 2. Human NSCLC samples	↑ p-Akt/Akt	[26]
miR-101	1. H157	? ATM	[27]
miR-Let-7c	1. H441	↓ p-STAT3	[28]
	2. H1975	↓ Ras/p-Akt	[29]

EGFR-TKIs, epidermal growth factor receptor-tyrosine kinase inhibitors; ↑, induction; ↓, repression; ?, unknown effect.

Restoration of gefitinib sensitivity has been observed in different NSCLC cell lines with chronic gefitinib exposure in response to miRNA stimulation. It has been reported recently that miR-34a reversed gefitinib resistance in HCC827 cells induced by concomitant incubation with hepatocyte growth factor (HGF) and gefitinib [20]. This concept is well-supported by the data showing that miR-34a restored apoptosis measured as percentage of apoptotic cells and cleaved PARP and reduced the protein abundances of both total and phosphorylated MET [20]. Consistent findings *in vivo* revealed that miR-34a induced regression of gefitinib-resistant tumours in conjunction with gefitinib intervention, an effect that could be ascribed to reduced expression of MET and phospho-EGFR^Tyr1068^ [20]. Although gene amplification of MET is only reported in 11% of the NSCLC patients [8], inhibition of MET by miR-34a may present a promising curative solution for these patients. Microarray analyses have identified that chronic gefitinib exposure increased the expression of 25 miRNAs while repressed 18 others in A549 clones; this differential expression profile was associated with a 3-fold increase in IC_50_ of gefitinib [21]. In line with the finding that miR-7 showed the most remarkable change in expression (*i.e.*, a 14-fold reduction) among other miRNA members studied, transfection with miR-7 abolished the elevation of EGFR and restored drug sensitivity in A549 cells exposed to long-term escalating doses of gefitinib [21].

The pro-apoptotic capacity of miRNAs has also been supported by data obtained from experimental models of acquired gefitinib resistance. Transfection with miR-138-5p mimics induced a dramatic sensitizing response to gefitinib in H1975 cells, which is in line with the observation that 6-month gefitinib exposure reduced miR-138-5p expression by 10 folds [30]. Incubation with miR-146a mimic elevated the number of apoptotic nuclei measured by PI fluorescent staining and activity of caspase 3/7 while mitigated phosphorylations of EGFR^Tyr1173^ and Akt^Ser473^ in H1975 cells, despite the fact that these effects were less pronounced relative to those induced by EGFR-targeting small-interfering RNA (siRNA) [22]. It is noteworthy that the total protein content of EGFR was concurrently reduced in response to miR-146a treatment, thus implying that EFGR is a modulating target of miR-146a [22]. Furthermore, the expression of miR-146a was ~63% lower in NSCLC samples compared with normal lung tissues whereas low expression of miR-146a was evident in patients with stage III/IV NSCLCs, thus pointing to the clinical significance of miR-146a in the progression of NSCLCs [22]. Recent evidence also showed that miR-146a induced direct repression on insulin receptor substrate 2 (IRS2), an adaptor protein essential in cell metabolism, at both transcript and protein levels [31]. Taken into consideration the fact that miR-146a expression was impaired in advanced lung cancer samples, strategies that inactivate metabolic/apoptotic signalling through the augmentation of endogenous level of miR-146a may re-sensitize patients to gefitinib therapy. Corroborating with the observation that the expression of miR-200c was reduced in clinical lung cancer specimens, the percentage of apoptotic cells was increased after over-expression of miR-200c in H460 cells [32]. These results imply that the capacity of miRNAs to manipulate the apoptotic pathway may provide high translational value to re-establish the therapeutic efficacy of EGFR-TKIs such as gefitinib.

The functional role of miRNAs in gefitinib resistance has also been characterized by epigenetic approaches. Experiments involving methylation-specific PCR analyses have demonstrated that the promoter of miR-9-3 was hypermethylated in H1975 cells [23]. Demethylation of miR-9-3 by 5-Aza-2′-deoxycytidine enhanced apoptosis measured as DNA fragmentation and caspase 3 activity induced by doxorubicin, which is a common prescription for breast cancer; these demethylation-related effects were antagonized by anti-miR-9-3 oligomer [23]. Previous efforts also indicated that the protein contents of Fas and anti-apoptotic Bcl2 were elevated and reduced respectively in H157 tumours expressing siRNA specific to the complement inhibiting protein CD59 [33], although whether the changes involved epigenetic regulation were unknown. Importantly, knocking down of CD59 in tumour bearing mice led to a survival rate of 70% compared with 0% in the counterparts with undisrupted CD59 expression [33]. The aforementioned findings therefore suggest that transcriptional repression by miRNAs may present a novel and effective therapeutic opportunity to NSCLC patients with EGFR-TKI resistance through the restoration of apoptosis as with direct target inhibition by siRNAs.

## 5. The Need of Clear, Functional Dissection of miRNAs

A growing body of opposing data suggests that not every miRNA would induce sensitizing drug response. Over-expression of miR-210 attenuated the expression of genes related to oxidative phosphorylation and induced enlargement of the mitochondria; these observations were coincident with the stabilization of hypoxia-inducing factor 1 alpha (HIF-1α) in A549 cells [24]. Further efforts elucidated that the increased stability of HIF-1α protein was followed by enhanced survival, although the p53 apoptotic pathway measured as p53, p21 and cleaved caspase 3 was activated with escalating dose of radiation [24]. Interestingly, the level of miR-150 was elevated in concomitant with reduction of SRCIN1 protein, which is an inhibitor of Src, in human lung cancer samples [25]. This inverse correlation was found to determine the migration capacity *in vitro*: transfection with miR-150 potentiated while forced expression of SRCIN1 alleviated cell migration [25]. In accordance with the observation that patients with high expression of miR-21 in NSCLC tissues manifested poor drug response and shortened overall survival, transcriptional silencing of miR-21 reduced tumour volume in mice implanted with gefitinib-resistant PC9 whereas forced-expression of miR-21 *in vitro* enhanced phosphorylation of Akt^Ser473^ and cell viability [26]. The notion that miR-21 mediates gefitinib resistance was confirmed further by the study demonstrating that intra-tumour administration with anti-miR-21 oligonucleotides antagonized the elevation of Akt in both total and phosphorylated forms in gefitinib-resistant PC9 tumours [34]. Experiments with H69AR, a cell line of small cell lung cancer, showed that miR-134, miR-379 and miR-495 were pivotal to the sensitizing response to various chemotherapeutics including cisplatin and doxorubicin, although whether perturbations of the miRNAs concerned would account for gefitinib resistance in NSCLC should merit further investigation [35]. In addition, it is also thought that the therapeutic efficacy of exogenous miRNAs would be confined by their intrinsic levels *in vitro*. A representative example of the claim would be that further stimulation with miR-101 in H157, a NSCLC cell line in which the expression of miR-101 is remarkably high, was not accompanied by reductions in surviving fraction and protein content of ATM (a target of miR-101 known to be involved in DNA repair) in response to ionizing radiation [27]. Similar results were replicated in *in vivo:* miR-101 blunted the growth of A549 and H1975 xenografts whereas the size of H157 tumours did not differ significantly with ectopic expression of miR-101 [27]. Intriguingly, miR-30c and miR-221/222 were robustly down-regulated after transcriptional inactivation of EFFR/MET in Calu1 cells, which is a gefitinib-unreceptive NSCLC cell line [36]. Subsequent analyses have demonstrated that these effects were only mimicked in gefitinib-sensitive PC9, but neither in Calu1 nor A549 after gefitinib incubation, therefore leading to the speculation that miR-30c and miR-221/222 may appear to be attractive intervention targets to reverse gefitinib resistance. However, it is not known how these miRNAs were regulated by EGFR and whether any synergism/antagonism exists among the miRNAs studied [36].

A growing body of evidence has proposed the use of miRNAs as markers of diagnosis and prognosis. In NSCLC patients, the expression of miR-21 in the serum was elevated and more importantly, patients with higher circulating levels of miR-21 had shorter survival time compared with those with lower levels [37]. This observation concurs with the inverse correlation between miR-21 expression in lung cancer tissues and response to gefitinib treatment [26]. Comparison among NSCLC patients has identified that the expression of miR-1260b was elevated by 2.7 folds in individuals with confirmed metastasis [38], suggesting that miR-1260b may modulate markers associated with the invasion process such as E-cadherin and vimentin as those reported with miR-520h [39]. Knocking down of miR-1620b consistently decreased migration activity of prostate cancer cells, an effect that could be at least in part, attributed to the negative regulation of Smad4 by miR-1620b, although whether this would also be a missing opportunity to regain sensitivity in gefitinib-resistant patients remains unknown [40]. A recent attempt has demonstrated the reliability of a miRNA signature consisting of miR-483-5p, miR-193a-3p, miR-25, miR-214 and miR-7, to characterize NSCLC patients in ethnically-diverse populations with success [41]. Notably, miR-7 was found to be elevated approximately 2-fold in sera from NSCLC patients compared to the control counterparts [41], which is contradictory to the observation that over-expression of miR-7 suppressed EGFR and induced sensitizing response to gefitinib *in vitro* [21]. In agreement with the observation that administration of miR-200c extended the survival of mice inoculated with H460, which is a NSCLC cell line known to express wild-type EGFR [32], the expression level of miR-200c was positively correlated with beneficial clinical outcome in advanced NSCLC patients receiving gefitinib/erlotinib therapies with no known EGFR mutations [42]. It is worth-noting, however, that this association was not found in NSCLC patients habouring EGFR mutations, despite the fact that a similar trend was observed [42], thus implying that the modes of action of miR-200c may be apparently distinct between conditions that are receptive/reluctant to gefitinib-induced cytotoxicity. It is certain that more research is necessary to determine the mechanisms underlying these inconsistent findings.

## 6. Synergy with Natural Phytochemicals

Emerging evidence also suggests that miRNAs may mediate the chemopreventive effects of natural, dietary compounds (Table 2) and thus may account for the reported changes in the epigenetic machineries and canonical PI3K/Akt survival signalling pathway as reviewed extensively elsewhere [43].

**Table 2:** Modulation of miRNAs by natural compounds.

Compounds of Interest	Changes in miRNA	Signalling Markers Involved	References
Antrocin	↑ miR-Let-7c	↓ Akt, JAK1/2, STAT3 ↑ Bax, cleavage of caspase 3	[28]
Resveratrol	↑ miR-335 ↑ miR-582-3p ↑ miR-338-3p ↑ miR194 and more	A number of genes related to apoptosis, cell cycle arrest and proliferation (Predicted)	[44]
	↑ miR-622	↓ k-Ras	[45]
	Augments the effects of miR-200c	↑ caspase 3/9, CHOP, p-JNK	[32]
	? miR-21	↑ Bcl2	[46]
	↓ miR-520h	↑ PP2A, E-cadherin ↓ p-Akt, FOXC2, vimentin	[39]
Curcumin	↑ miR-192-5p, miR-215	↑ p53, p21	[47]
	↓ miR-186	↑ caspase 10	[48]

Antrocin is one of the most abundant small molecules of medicinal mushroom believed to confer preventive effects against the development of cancer. Recent attempts have demonstrated that antrocin mitigated phosphorylations of various survival-related kinases including Akt^Ser473^, JAK1/2^Tyr1002/Tyr1007^ and STAT3^Tyr705^ and stimulated apoptosis indicated by increased expression of pro-apoptotic Bax and cleavage of caspase 3 and down-regulation of anti-apoptotic Bcl2 in H441 cells [28]. What is of higher importance is that all these observations coincided with an elevated abundance of miR-Let-7c, suggesting that miR-Let-7c might promote cell death in NSCLCs [28]. This postulation is confirmed by the experiments showing that transient induction of miR-Let-7c ameliorated the activity of STAT3 and formation of colonies, in which all these effects were amplified by combination treatment with antrocin [28]. Compelling data also documented that forced expression of miR-Let-7c in H1975 cells restored the cytotoxicity of gefitinib, an effect observed concomitantly with decreases in protein contents of oncogenic Ras and phosphorylated Akt^Ser473^ [29]. Recent attempts to enhance the effect of erlotinib with combinations of miR-Let-7c and miR-34 were of a great success [49]. Extrapolation of the aforementioned findings to natural phytochemicals is apparently an important scope of research, provided that the concepts of off-target symptoms of erlotinib and association between miR-Let-7c and shortened survival in lung cancer patients are well-established [50].

Previous efforts have also shown that resveratrol, which is a natural antioxidant in grapes and red wine, modulated an array of miRNAs in A549 cells. In particular, the expression of various miRNA members changed abruptly with resveratrol stimulation (miR-335: 19.87-fold; miR-582-3p: 21.4-fold; miR-338-3p: 36.59-fold; miR194: 42.91-fold, *etc.*) [44]. Using bioinformatics analysis, a panel of genes related to apoptosis, cell-cycle arrest, growth and proliferation were predicted to be targets of the aforementioned miRNAs [44]. MicroRNA profiling analyses have indicated that the content of miR-622 was robustly elevated by resveratrol incubation in cultures of H460 and 16HBE-T, which is a transformed form of the bronchial epithelial cell line 16HBE [45]. In concordance with the diminished viability in resveratrol-treated 16HBE-T cells, increased cell cycle arrest and blunted proliferation were observed in both cell lines with transient up-regulation of miR-622 [45]. It is also noteworthy that resveratrol did not perturb the viability of normal epithelial cells and mice transfected with miR-622 manifested lighter tumours upon challenge with 16HBE-T inoculation, hence bringing forward the possibility that the manipulation of miR-622 by resveratrol may elicit specific therapeutic outcomes in lung cancer patients with minimal undesired effects [45]. Given that transcriptional repression of Bcl2 was provoked secondary to the resveratrol-induced decrease in miR-21expression in *in vitro* models of pancreatic cancer [46] whereas miR-21 has been reported to be associated with poor drug treatment outcomes in lung cancer patients [26], it is tempting to investigate whether the miR-21/Bcl2 axis would represent a mechanism of action of resveratrol in the combat against drug resistance in NSCLC. Recent attempts have proposed a critical role formiR-520h during lung cancer metastasis, by which its over-expression antagonized the stimulation of PP2A by resveratrol, leading to the reversion of a high phosphorylation level of Akt and ultimately, the migrating capacity of A549 cells [39]. All these studies support the speculation that pharmacological activation/inactivation of endogenous, clinically-relevant miRNAs by resveratrol may revert the sensitivity of lung cancer tissues to gefitinib therapy.

Curcumin, a bioactive ingredient in curry, has been demonstrated to elevate significantly the contents of miR-192-5p and miR-215 concomitantly with pro-apoptotic p53 and cell-arresting p21 in H460 cells [47]. Experiments with A549 cells unraveled that curcumin up-regulated caspase 10 robustly and repressed proliferation whereas these findings were recapitulated with the inhibition of miR-186 [48]. Of note, similar findings were observed with forced expression of a miR-186 inhibitor in A549/DDP multidrug-resistant human lung adenocarcinoma cells, thereby confirming the oncogenic nature of miR-186 [51]. Transfection with miR-21 mimic in A549 cells was found to abrogate the anti-proliferative/anti-apoptotic effects of curcumin as determined by MTT assay and annexin-V/PI staining respectively [52]. Corroborating data obtained from pancreatic cancer cells have also revealed that resveratrol increased the transcript contents of various tumour suppressors known to be regulated negatively by miR-21 [53]. Taken into consideration the fact that the negative correlation between miR-21 expression and relapse-free survival in NSCLC patients is well-documented [54], these studies provide an important insight that pharmacological blockade of miR-21 by curcumin/resveratrol may improve the prognosis with gefitinib treatment. Activation of caspase 3 and PARP fragmentation, which are markers indicative of apoptosis, were observed in A549 cells following over-expression of miR-192-5p and miR-215 whereas these findings were reproduced with curcumin incubation [47]. Furthermore, the induction of miR-192-5p and miR-215 by curcumin required the presence of functional p53 protein, hence leading to the thought that the anti-tumour effects of curcumin and its miRNA targets may orchestrate through a positive-feedback manner [47].

Nevertheless, it remains largely unknown whether the therapeutic values of these natural compounds would be augmented by simultaneous treatment with their putative miRNA mimetics. An encouraging study has shown that combined injection of resveratrol and miR-200c prolonged the survival of H460-bearing mice more remarkably than either treatment alone; by which this effect could be in part ascribed to the increases in caspase 3, caspase 9 and CHOP [32]. Taken together, these studies shall empower future research to dissect the synergistic/antagonistic mechanisms between miRNAs and natural compounds to remedy gefitinib-resistant NSCLCs, a clinically-significant challenge in lung cancer.

## 7. Conclusions

**Figure 1:**
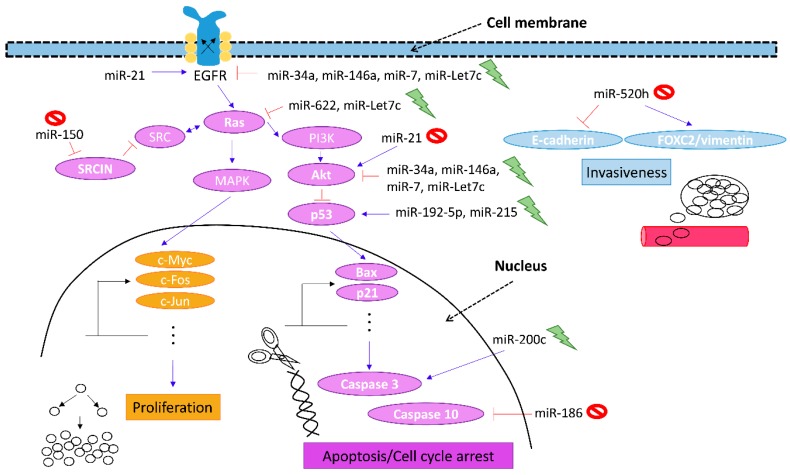
Plausible mechanisms of action of miRNAs in the restoration of gefitinib sensitivity. Incompetent repression of epidermal growth factor receptor (EGFR) is a molecular hallmark of gefitinib resistance. Hence, targeting signalling markers downstream of EGFR may provide novel therapeutic opportunity for NSCLC patients with acquired gefitinib resistance. Intense research efforts have demonstrated the capacity of miRNAs in the modulation of a number of signalling molecules related to growth/proliferation (Ras and SRCIN), apoptotic cell death/cell cycle arrest (p53, Bax, caspases and p21) and cell invasion (E-cadherin and vimentin) in conjunction with the EGFR phosphorylation cascade (Akt), which may contribute to the reversion of gefitinib sensitivity. Blue arrow, stimulation; red “T” sign, inhibition; dashed arrows, indication of cellular components.

Whilst overcoming the mutations causing gefitinib resistance in NSCLC patients are apparently infeasible at the present moment, researching molecules associated with proliferation, survival and cell death offers an alternate approach for biomarker discovery and the development of novel therapeutic regimens. A growing body of data points out that various miRNAs impede the growth of gefitinib-resistant tumours both *in vitro* and *in vivo*, meaning these effects can be attributed largely to the manipulation of EGFR/MET pathway and apoptotic cell death (Figure 1). Recent studies also suggest that the chemopreventive effects of various natural dietary compounds may involve the modulation of miRNAs. Nevertheless, whether combined therapy with phytochemicals and miRNAs would provoke more pronounced anti-cancer effects warrants further investigation.

## Abbreviations

Akt	Protein kinase B
AMPK	AMP-activated protein kinase
ATM	Ataxia telangiectasia mutated
ATP	Adenosine triphosphate
CD31	Cluster of differentiation 31
EGF(R)	Epidermal growth factor (receptor)
EGFR-TKI	Epidermal growth factor receptor tyrosine kinase inhibitor
HIF-1α	Hypoxia-inducing factor 1 alpha
HGF	Hepatocyte growth factor
IC_50_	Half maximal inhibitory concentration
IGFR	Insulin growth factor receptor
JAK1/2	Janus kinase 1/2
MET	Hepatocyte growth factor receptor
miRNA	MicroRNA
mTOR	Mammalian target of rapamycin (mTOR)
NSCLC	Non-small cell lung cancer
PARP	poly ADP-ribose polymerase (PARP)
PCNA	Proliferating cell nuclear antigen
PDH	Pyruvate dehydrogenase
PI3K	Phosphatidylinositol-3-kinase
PP2A	Protein phosphatase 2
RISC	RNA-induced silencing complex
ROS	Reactive oxygen species
siRNA	Small-interfering RNA
SRCIN1	SRC kinase signalling inhibitor 1
STAT3	Signal transducer and activator of transcription 3
VEGF	Vascular endothelial growth factor
UTR	Untranslated region
